# Histamine Modulates Midbrain Dopamine Neuron Differentiation Through the Regulation of Epigenetic Marks

**DOI:** 10.3389/fncel.2019.00215

**Published:** 2019-05-21

**Authors:** Fernanda Vargas-Romero, Rodrigo González-Barrios, Lissania Guerra-Calderas, Itzel Escobedo-Avila, Daniel Cortés-Pérez, Adolfo López-Ornelas, Luisa Rocha, Ernesto Soto-Reyes, Iván Velasco

**Affiliations:** ^1^Instituto de Fisiología Celular – Neurociencias, Universidad Nacional Autónoma de México, Mexico City, Mexico; ^2^Unidad de Investigación Biomédica en Cáncer, Instituto Nacional de Cancerología-Instituto de Investigaciones Biomédicas, Universidad Nacional Autónoma de México, Mexico City, Mexico; ^3^Departamento de Ciencias Naturales, Universidad Autonoma Metropolitana, Unidad Cuajimalpa, Mexico City, Mexico; ^4^Laboratorio de Reprogramación Celular, Instituto Nacional de Neurología y Neurocirugía “Manuel Velasco Suárez” – Instituto de Fisiología Celular, Universidad Nacional Autónoma de México, Mexico City, Mexico; ^5^Departamento de Farmacobiologia, Centro de Investigación y de Estudios Avanzados (Cinvestav), Mexico City, Mexico

**Keywords:** midbrain development, dopamine neuron differentiation, epigenetic modifications, neuroepigenetics, ultrasound-guided injection in utero

## Abstract

During midbrain development, dopamine neuron differentiation occurs before birth. Epigenetic processes such as DNA methylation and demethylation as well as post-translational modification of histones occur during neurogenesis. Here, we administered histamine (HA) into the brain of E12 embryos *in vivo* and observed significant lower immunoreactivity of Lmx1a+ and Tyrosine Hydroxylase (TH)+ cells, with parallel decreases in the expression of early (*Lmx1a*, *Msx1)* and late (*Th*) midbrain dopaminergic (mDA) genes. With MeDIP assays we found that HA decreases the percentage of 5-methylcytosine of *Pitx3* and *Th*, without changes in 5-hydroxymethylcytosine. Additionally, HA treatment caused a significant increase in the repressive epigenetic modifications H3K9me3 in *Pitx3* and *Th*, and also more H3K27me3 marks in *Th*. Furthermore, HA has a long-term effect on the formation of the nigrostriatal and mesolimbic/mesocortical pathways, since it causes a significant decrease in midbrain TH immunoreactivity, as well as alterations in dopaminergic neuronal fibers, and significant lower TH-positive area in the forebrain in whole-mount stainings. These findings suggest that HA diminishes dopaminergic gene transcription by altering several epigenetic components related to DNA and histone modifications, which affects mDA neuron progression during development.

## Introduction

Midbrain dopaminergic (mDA) neurons are essential for motor function control as well as for the regulation of reward and emotions (van Heesbeen et al., 2013). Development of mDA neurons can be divided into three stages: neural plate regionalization, midbrain cell fate determination and terminal differentiation to mDA neurons (Gale and Li, 2008). Each step presents activation of different cascades of transcriptional factors triggered by extrinsic signals (Ang, 2006; Abeliovich and Hammond, 2007; Huang et al., 2015). During the specification stage, dopaminergic neural progenitors express specific genes such as *Lmx1a/b*, *Foxa2*, *Msx1/2*, and *Ngn2* (Ang, 2006; Gale and Li, 2008; Nakatani et al., 2010; Yan et al., 2011). At this stage neural progenitors acquire mDA neuronal fate and start the last stage to become post-mitotic neurons (Yi et al., 2014). The activation of *Nurr1* and *Pitx3* allows the expression of late-differentiation genes associated to the dopaminergic neuron phenotype, as they regulate the expression of enzymes such as Tyrosine Hydroxylase (TH), the rate limiting enzyme in the production of dopamine (Gale and Li, 2008; Jacobs et al., 2009; Kadkhodaei et al., 2009). Terminally differentiated mDA neurons extend their axons to reach the *striatum* (ST) and release dopamine to regulate motor function in the basal ganglia (Hegarty et al., 2013).

Histamine (HA) is a neurotransmitter/neuromodulator that participates in the sleep/awake cycle, motor activity, cognition, feeding and energy balance in the adult brain (Panula and Nuutinen, 2013). During rat embryonic development, the cerebral concentration of HA reaches higher levels than those observed in the adult; its highest concentration is found at embryonic days (E) 14–16 (Vanhala et al., 1994; Nissinen and Panula, 1995; Molina-Hernández et al., 2012). This amine has a cell proliferation and neuronal differentiation effect on cortical neural stem/progenitor cell (NSPC) cultures, through multiple effects, including: elevation of intracellular Ca^2+^, up-regulation of FGF receptors and increased expression of proneural genes (Molina-Hernández and Velasco, 2008; Rodríguez-Martínez et al., 2012; Molina-Hernández et al., 2013). Similar effects of HA, have been observed on adult NSPC at the subventricular zone (Bernardino et al., 2012). In addition, the *in vivo* effects of HA injected at E12 include a decrease in NSPC proliferation in the ventricular zone and also a decrease in TH staining in the midbrain, without affecting serotoninergic nor GABAergic neurons; co-injection of HA with the H_1_R antagonist chlorpheniramine prevented the decrease on TH induced by HA (Escobedo-Avila et al., 2014).

Recent studies describe different pathways to regulate neural stem cell differentiation via regulation of epigenetic modifications, which result in changes on gene expression. DNA demethylation has been related to the brain development control (Hahn et al., 2013; Wheldon et al., 2014). Particularly, the increase of 5-hydroxymethylcytosine (5hmC) along gene bodies has been associated with transcriptional activation during neuronal differentiation (Hahn et al., 2013; Kim et al., 2014). Furthermore, abundance of 5-methylcytosine (5mC) on gene body regions has been related to up-regulation of pre-mRNA splicing (Shukla et al., 2011; Guo et al., 2014; Yearim et al., 2015). These and other epigenetic modifications can be induced by environmental stimuli (Aguilera et al., 2010; van Heesbeen et al., 2013). For example, long-term exposure of cultured ventral midbrain (VM) NSPC to depolarizing potassium concentrations promotes mDA neuron differentiation, increasing binding of Nurr1 to the regulatory regions, and higher transcriptional activity, of *Th* and *Dat* genes, with parallel increases in H3K4me3 and decreased H3K9me3/H3K27me3, although the latter changes observed only in the *Th* promoter (He et al., 2011). Furthermore, VM NSPC treated with the neuropeptide urocortin increases mDA differentiation by inhibition of Histone Deacetylase 1 (HDAC1), resulting in hyperacetylation of histone H3, which allows the binding of Nurr1 to the upstream *Th* regulatory regions (Huang et al., 2015). This suggests that post-translational histone modifications participate in the regulation of the expression of genes involved in brain development.

In this work, we aimed to evaluate the effect of HA on several epigenetic marks on intragenic regions that regulate expression of genes involved in mDA development *in utero*, as well as its long-term effects *in vivo* before birth. We found an enrichment of repressive histone marks, H3K9me3 and H3K27me3, together with a decrease of 5mC at intergenic regulatory regions. Such modifications were associated to significantly lower mRNA levels of genes related to midbrain DA neuron fate specification. Injection of HA at E12 caused a decrease in the number of mDA neurons and modified the trajectory of its axons to the ST at late stages of midbrain development.

## Materials and Methods

### Ultrasound-Guided Injections

All animal procedures were approved by the Institute of Cellular Physiology’s Animal Care and Use Committee and conformed to National guidelines (*Norma Oficial Mexicana* NOM-062-ZOO-1999). We used timed-pregnant Wistar rats with embryos at gestational age E12 to perform ultrasound-guided injections as previously reported (Escobedo-Avila et al., 2014). Control (vehicle-injected) or experimental (HA-injected) conditions were performed by triplicate or quadruplicate in different embryos from the same rat, and repeated with six different pregnant dams. Each group was evaluated in a single uterine horn. Rats were deeply anesthetized in a chamber and then maintained with a facemask to administer inhaled sevoflurane (5% for induction and 1% for maintenance). We performed a midline laparotomy, the uterine horns were carefully exposed and the number of embryos was recorded. To visualize the embryos, we used an MHF-1 Ultraview UltraSound System (E-Technologies) with a focal distance of 7 mm. Intraventricular injections were performed through the uterine wall using pulled microcapillaries (borosilicate glass, Sutter Instruments). The glass needles were visualized in the ultrasound imaging system and when in the lateral ventricles, the injection of 2 μl was verified by observing the liquid going out of the needle with the aid of an automatic injector (Quintessential injector, Stoelting). Control embryos received injectable water and experimental embryos were injected with 50 μg of HA dihydrochloride (Sigma-Aldrich), a dose previously determined to decrease TH in the MB (Escobedo-Avila et al., 2014). The osmolarity of this HA solution was 348 mOsm, and HA decreased 0.6 pH units in relation to the vehicle. We have showed (Escobedo-Avila et al., 2014) that the injection of vehicle, or HA at this concentration, did not cause morphological abnormalities in the MB, ruing out damage caused by the osmolarity or pH. After injection, the uterus was repositioned in its physiological site. The incisions of the abdominal muscle and skin were stitched with separate sutures. Finally, we applied Buprenorphine as analgesic (0.1 mg/kg, Pisa Laboratories) and animals were monitored and left to recover. Two or 6 days after the injections, pregnant rats were euthanized by an overdose of sodium pentobarbital (Pfizer), and identified embryos were recovered for analysis. We performed the following number of successfully injected E12 embryos: vehicle: 53; HA: 59. The number of dams used was 22.

### Immunohistochemistry

Embryos were removed and transferred to PBS. Subsequently fixed by immersion in 4% paraformaldehyde solution in PBS at 4°C, overnight (for E14 embryos) and for 6 days (for E18 embryos). Afterward, tissues were cryoprotected overnight in 30% (v/v) sucrose, embedded in Tissue Freezing Medium (Tissue-Tek, Zakura Finetek) and frozen at −80°C. Coronal and sagittal sections of 20 μm were obtained on a cryostat (Leica). For immunohistochemistry, slides were rehydrated in PBS. Tissue sections were treated with immuno-retriever for 30 min at 65°C and subsequently, permeabilized and blocked for 1 ½ h at room temperature with 0.3% Triton X-100 and 5% horse serum in PBS. Slides were incubated overnight at 4°C with the following primary antibodies diluted in PBS containing 5% horse serum: rabbit polyclonal anti-TH (1:1000, Pel-freez), rabbit polyclonal anti-Lmx1a (1:1500, Millipore), mouse monoclonal anti-β-III Tubulin (1:500, Babco Covance). Then, the sections were washed three times for 5 min in PBS and incubated with the secondary antibodies Alexa-Fluor 488 anti-rabbit IgG and Alexa-Fluor 568 anti-mouse IgG (1:500; Molecular Probes) in PBS containing 5% horse serum for 2 h. Nuclei were stained with Hoechst 33258 (1 ng/mL). After three further washes in PBS, the sections were mounted in Aqua Poly/Mount (Polysciences, Inc.) Immunostainings were analyzed with an epifluorescence microscope (Nikon, Eclipse TE2000-U) and photographed with a Nikon digital camera (DMX1200 F). Some images were acquired with a FV1000 Olympus confocal microscope. Negative controls were performed in the absence of primary antibodies and showed no unspecific staining.

### Immunohistochemistry of Whole-Mount Brains From Embryos

We perform immunohistochemistry of whole-mount brain as previously reported (Joyner and Wall, 2008). Embryos at E18 stage were incubated in 20% dimethyl sulfoxide (DMSO): 80% methanol (fixative solution) for 24 h at 4°C. Then, the embryos were transferred to 10% hydrogen peroxide diluted in fixative and incubated overnight at room temperature. This treatment effectively bleached the pigment of the embryo and blocked endogenous peroxidase activity. After bleaching, embryos were incubated in 100% methanol at −20°C for 4 h. For staining, bleached embryos were washed twice for 1 h with 2% skim milk and 0.5% Triton X-100 in PBS (PBSMT). Embryos were incubated overnight at 4°C with rabbit polyclonal anti-TH (1:1000, Pel-freez) in PBSMT. After this incubation, the embryos were washed seven times in PBSMT; each wash lasted 1 h. The embryos were then incubated overnight at 4°C with affinity-purified goat anti-rabbit immunoglobulin antibody conjugated to horseradish peroxidase (1:500, Santa Cruz) in PBSMT. The seven washes were repeated. Bound peroxidase-conjugated antibody was visualized using the DAB Substrate Kit for Peroxidase (VECTOR laboratories); reactions were carried out for 10 min at room temperature. Embryos were post-fixed in 4% paraformaldehyde in PBS at 4°C overnight and dehydrated in 50, 80, and 100% methanol (1 h each). Embryos were cleared by placing them in a 1:2 mixture of benzyl alcohol:benzyl benzoate. Immunostainings were visualized with a stereomicroscope (Nikon, SMZ1500) and photographed with a Nikon digital camera (Coolpix S10). This preparation allows to visualize the nigro-striatal as well as the mesolimbic and mesocortical pathways. We measured the area of TH+ staining with ImageJ software in: (a) the midbrain (including the *substantia nigra pars compacta* and the Ventral Tegmental Area); (b) the Medial Forebrain Bundle (MFB); and (c) the forebrain (striatum/cortex, including the Nucleus Accumbens). The average of four embryos was determined per treatment. Analysis is presented as the mean ± standard error of the mean (SEM) and statistical analysis by multiple *t*-test. Since the immunostaining with positive TH was tridimensional, the area of positive label was the preferred parameter to measure.

### Quantitative RT-PCR (RT-qPCR) Analysis

Total RNA was extracted with TRIzol (Invitrogen) according to the manufacturer’s specifications. The mRNA levels were determined by RT-qPCR performed with cDNA generated from 2 μg of total RNA using the Kit GeneAmp RNA PCR KIT (Applied Biosystems). Primers were synthetized based on the UCSC Genome Browse database from *Rattus norvegicus* (Gibbs et al., 2004; Havlak et al., 2004). Gene expression levels were determined using the following primers (all listed in 5′to 3′ orientation; Sense, S; Antisense, AS): *Lmx1a*, S: GCACGGAAGCTAGACTCAA; AS: GCTCTGCCCAGCAAAGAG; 143 bp. *Msx1*, S: CTGTTGGGGGACTCCTCAA; AS: GCCGCCTGGCTGGGGG; 119 bp. *Foxa2*, S:CAGAAAAAGGCCTGAGGTG; AS: CAGCATACTTTAACTCGCTG; 137 bp. *Nurr1*, S: GTCACAGAGAGACACGGG; AS: GGTAGTTGGGTCGGTTCAA; 121 bp. *Pitx3*, S:CTCGAAGCCCTGCGCTGT; AS: GCCTTCTCCGAGTCACTGT; 100 bp. *Th*, S: CCACTGGAGGCTGTGGTATT; AS: CCGGGTCTCTAAGTGGTGAA; 145 bp. H_1_ receptor,S: CTTCTACCTCCCCACTTTGCT; AS: TTCCCTTTCCCCCTCTTG; 292 bp. H_2_ receptor S: TTCTTGGACTCCTGGTGCTGC; AS: CATGCCCCCTCTGGTCCC; 309 bp. *Gapdh*, S: GTGGACCTCATGGCCTACAT; AS: GGATGGAATTGTGAGGGAGA; 160 bp. *Gapdh* was used as an internal control. The qPCR reactions for 96-well plate format were performed using 50 ng of cDNA and the Thermo Maxima SYBR Green/ROX 1 PCR Master Mix (Thermo Fisher Scientific) with a StepOnePlus Real-Time PCR System (Applied Biosystems). The fold change was calculated by the 2^−ΔΔCt^ method (Livak and Schmittgen, 2001).

### Sodium Bisulfite Sequencing

Genomic DNA was isolated by phenol:chloroform:isoamyl alcohol (Sigma-Aldrich) extraction technique as reported (Clark et al., 2006). One microgram of genomic DNA was modified by sodium bisulfite using the EZ DNA methylation kit (ZYMO Research). For sequencing, modified DNA was amplified by PCR using the following primers recognizing *Th*, S: GTAGGTGTTTGTGATAGTGGATG; AS: AAAACCACTCCAACCCTAAATA, with a length of 439 bp. These primers were synthetized based on the UCSC Genome Browse database from *R. norvegicus* (Gibbs et al., 2004; Havlak et al., 2004). The PCR products were purified and cloned using the pGEM-T Easy Vector system (Promega). Eight clones were randomly chosen and sequenced individually. The percentage of modified cytosines was calculated for vehicle- and HA-treated embryos.

### Methylated and Hydroxymethylated DNA Immunoprecipitation Assay(MeDIP and hMeDIP)

Genomic DNA was isolated as described above and processed by ultrasonic pulses (GENEQ, GEX500; Cole-Parmer). Initial experiments were set up to obtain fragments of an average size of 300 bp. Such conditions included 3 rounds of pulses of 30 s, with sequences of pulse-on for 5 s and pulse-off for 3 s at 35% amplitude. DNA was immunoprecipitated using a MeDIP/hMeDIP kit (Diagenode). Briefly, 1 μg of fragmented DNA was denatured for 3 min at 95°C and incubated with 2.5 μg of the company-validated mouse monoclonal anti-5 hmC (Diagenode) or 2 μg of mouse monoclonal anti-5mC (Diagenode). The antibody-bound DNA was recovered by immunoabsorption with anti-mouse IgG-coated magnetic beads (Diagenode) for 16 h at 4°C. The immunoprecipitated DNA was amplified by 30 cycles of PCR. Primers were designed from the sequences obtained in the UCSC Genome Browse database from *R. norvegicus* (Gibbs et al., 2004; Havlak et al., 2004). For the mDA differentiation genes, oligonucleotides were designed in exons, which represent regulatory regions for neurogenesis. Exons were chosen based on their higher percentage of CpG content or within a CpG island. Primers used are as follows: *Lmx1a*, exon 2, S: GGGTCATCTCGGATAGGTTT; AS: CAGGCACTTACTTCTCGTAG; 156 bp. *Msx1*, exon 1,S: CGAAACCCATGATCCAGGG; AS: GTCCTCCACTTTGACACCG; 112 bp. *Foxa2*, exon 3, S: CGCTCATCACCATGGCCAT; AS: GCCGGTAGAAAGGGAAGAG; 96 bp.*Nurr1*, exon 2, S: CAGTCCGAGGAGATGATGC; AS: GGAAGTTGTGAAGGGAGCC; 133 bp. *Pitx3*, exon 4, S: CTTAGTCCCTGCCAGTACG; AS: GCACCCCTTTTCAGACCCT; 149 bp. *Th*, exon1, S: GAGACAGAACTCGGGACC; AS: CGGGTGACAGCATATCCTC; 145 bp. The efficiency of MeDIP or hMeDIP products of a particular genomic locus was calculated from qPCR by a fast optical 96-well q PCR reaction plate (Applied Biosystems). The qPCR reaction was performed using Thermo Maxima SYBR Green/ROX 1 PCR Master Mix (Thermo Fisher Scientific) with a StepOnePlus Real-Time PCR System (Applied Biosystems). According to the manufacturer inset, data for each sample is reported as recovery of starting material: % MeDIP or hMeDIP/INPUT.

### Chromatin Immunoprecipitation to Detect H3K9me3 and H3K27me3

Ventral midbrains were dissected and pooled from 7 embryos treated with HA or vehicle. Tissue was maintained in ice-cold PBS before crosslinking, which was carried out for 10 min, with 1% of formaldehyde. The crosslink reaction was stopped for 5 min with 125 mM glycine and the crosslinked tissue was washed three times with ice-cold PBS for 5 min. After the last wash, tissue was lysed with 500 μL of lysis buffer. Chromatin was sonicated using an Ultrasonic Processor (GENEQ, GEX500; SOVC505-00). After sonication, DNA was extracted and its length evaluated. Quantitation of chromatin was made by the Lowry reaction and 100 μg of material was used per IP. Immunoselection was performed according to the protocol of the OneDay ChIP kit (Diagenode, Kch-oned IP- 180), with anti-H3K9me3 or anti-H3K27me3 (Diagenode). Immunoprecipitation was performed by incubating 20 μL of magnetic beds (16-663 | Magna ChIP^TM^ Protein A+G Magnetic Beads), with the antibody-chromatin complexes for 3 h in a rotating wheel; then the beads-antibody-chromatin complexes were washed three times with ice-cold ChIP buffer 1×. Finally, the DNA was decrosslinked overnight and purified employing the MinElute Reaction Cleanup Kit (Cat No./ID: 28204) following the manufacturer’s instructions. ChIP assays were performed by qPCR in a fast optical 96-well qPCR reaction plate (Applied Biosystems) with the same primers used for MeDIP/hMeDIP for *Pitx3* and *Th*. The qPCR reaction was performed using Thermo Maxima SYBR Green/ROX1 PCR Master Mix (Thermo Fisher Scientific) with a StepOnePlus Real-Time PCR System (Applied Biosystems), according to the manufacturer’s instructions.

### Dopamine Quantification by High-Performance Liquid Chromatography (HPLC)

E18 embryos were recovered after vehicle or HA injection at E12. The striatum was dissected and individually homogenized with a mixture of 0.1 M perchloric acid (Baker), 4 mM Na_2_S_2_O_3_ (Sigma), 0.1 mM EDTA (Sigma). The homogenate was centrifuged (12,600 rpm, 20 min at 4°C). Subsequently, the supernatant was filtered with 0.45 μm filters (Millex-HV) and 20 μl were mixed with 7.5 μL of antioxidant solution (0.1 N perchloric acid; 0.02% EDTA and 1% EtOH) and injected into the solvent stream of a HPLC system, using a reversed-phase column (C18, 3 μm; 2.1 × 50 mm; Atlantis, Waters) coupled to a pre-column (Nova-Pack, Waters) with a mobile phase solution containing 0.054 mM EDTA, 50 mM citric acid, and 0.1 mM octansulfonic acid, dissolved in milli-Q water and mixed with methanol in a proportion of 97:3, respectively. The pH was 2.9 and the flow rate 0.35 mL/min. Dopamine detection was performed by a single-channel electrochemical amperometric detector (Waters model 2465) at 450 mV at a temperature of 30°C, and quantified by peak height measurements against standard solutions with know dopamine concentration, as described (Rocha et al., 2012).

### Statistical Analysis

Data from four to six independent experiments are expressed as the mean ± SEM. The differences between groups were analyzed using the unpaired Student’s *t*-test comparing the HA- with the vehicle-treated rat embryos. Differences between groups were considered statistically significant when *p* < 0.05.

## Results

### Intrauterine Administration of HA Decreases TH and Lmx1a Immunoreactivity in the VM

**FIGURE 1:**
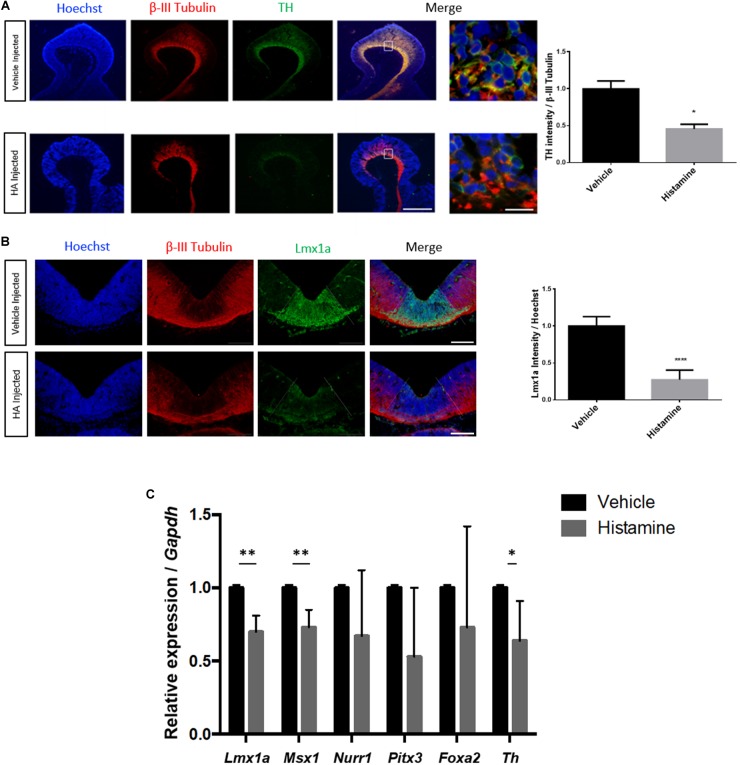
HA decreases TH and Lmx1a immunoreactivity and down-regulates dopaminergic genes in the ventral midbrain (VM) after *in utero* injection. Rat embryos were injected at E12 and analyzed 2 days later. **(A)** Immunohistochemistry showing a significant decrease, caused by HA, in the DA marker TH, in sagittal sections. The neuronal marker β-III Tubulin was unaffected by HA, and was used to normalize TH immunoreactivity. Nuclei were detected with Hoechst. The white square in the merge image is expanded on the right. Scale bar: 150 μm in the low-magnification and 10 μm in the amplification. **(B)** HA significantly reduces the area positive for the dopaminergic progenitor marker Lmx1a, without affecting β-III Tubulin. The quantification of Lmx1a intensity was normalized by the Hoechst signal. The area of Lmx1a-positive DA progenitor cells is delimited by the dotted lines and was dissected to perform RT-qPCR. Scale bar: 200 μm. **(C)** Expression of genes involved in DA differentiation after intrauterine HA or vehicle administration, analyzed at day E14. Data was normalized by *Gapdh* expression. Vehicle-treated embryos were used as the reference for the 2ˆ–ΔΔCt analysis. Graphs represent the mean values with SEM from 3 **(A,B)** to 5 **(C)** independent experiments. ^∗^*p* < 0.05, ^∗∗^*p* < 0.01, ^∗∗∗∗^*p* < 0.0001.

We performed *in utero* microinjections of HA or vehicle, into the ventricular lumen close to the MB of E12 rat embryos, allowing them to develop to E14. Because it was previously reported that intrauterine injection of HA at E12 precludes mDA differentiation at E14 (Escobedo-Avila et al., 2014), we quantified the immunoreactivity of TH and Lmx1a in vehicle- or HA-injected embryos, confirming a significant decrease in TH (Figure 1A). We also assessed the effect of HA on VM dopamine progenitors positive to Lmx1a (Nakatani et al., 2010; Yan et al., 2011). We found that HA down-regulates Lmx1a reactivity, without altering β-III Tubulin, in contrast to the vehicle-treated embryos (Figure 1B).

### Administration of HA Down-Regulates the Expression of Specification and Differentiation Dopaminergic Genes

Based on the Lmx1a-positive domain, we performed dissections selecting the VM of injected embryos 2 days after surgery. Subsequently, we analyzed the expression of DA specification (*Lmx1a, Msx1, and Foxa2*) and differentiation (*Nurr1, Pitx3, and Th*) genes by RT-qPCR. After normalization with *Gapdh*, we found that HA significantly decreases the expression of *Lmx1a*, *Msx1*, and *Th* compared to vehicle-injected embryos (Figure 1C). H_1_ and H_2_ receptors are expressed in the VM; we did not find significant changes in these receptors’ expression after HA injection (data not shown).

### HA Reduces 5mC at the Exons Regions of Genes Associated With DA Differentiation

**FIGURE 2:**
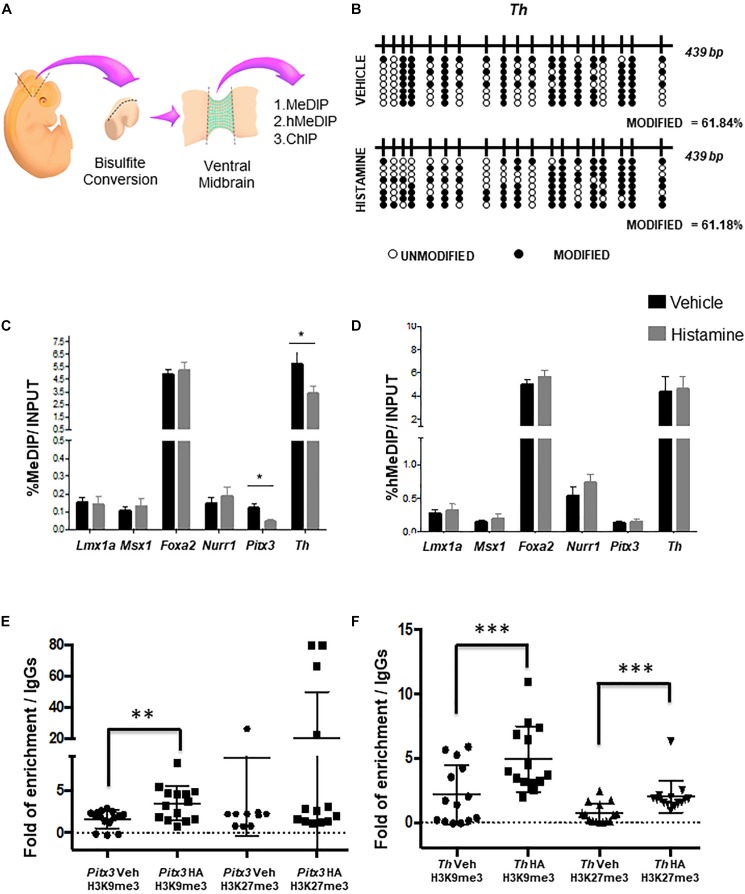
Epigenetic modifications caused by HA *in utero*. **(A)** Scheme of the experimental design to dissect the midbrain and its ventral portion delimited by Lmx1a-positive cells, for the indicated analyses. **(B)** No changes were detected on modified cytosines, using sodium bisulfite and sequencing after HA administration in the MB, 2 days after intrauterine injection. Black circles represent modified CpGs, and white circles represent unmodified CpGs. **(C,D)** Enrichment analysis of 5-methylcytosine and 5-hydroxymethylcytosine in different genes involved in DA differentiation. qPCR analysis on DNA after immuno-precipitation with anti-5mC (**C**, MeDIP) and anti-5hmC (**D**, hMeDIP) antibodies in HA- or vehicle-injected embryos. Internal negative and positive controls were performed according to the manufacturer’s specifications (Diagenode; mc-magme-048). Data was normalized against 10% INPUT. Chromatin immunoprecipitation analysis of H3K9me3 and H3K27me3 levels in *Pitx3* **(E)** and *Th* **(F)** genes. The box plots represent the qPCR amplifications of the fourth exon of *Pitx3* and the first exon *Th*, using the DNA obtained from the ChIP assay employing anti-H3K9me3 and anti-H3K27me3 antibodies, in tissue treated with either vehicle or HA. As a negative control, we used the IgG antibody included in the OneDay ChIP kit (Diagenode, NJ, United States, Kch-onedIP-180). ^∗^*p* < 0.05, ^∗∗^*p* < 0.01, ^∗∗∗^*p* < 0.005 compared to the vehicle-treated tissue from 3 **(B)** to 4 **(C–F)** independent experiments.

Next, we asked if HA affected epigenetic modifications associated to DNA. By bisulfite sequencing, we evaluated the DNA methylation patterns in HA-treated embryos, after MB dissections (Figure 2A). We did not observe overall changes in the percentage of modified cytosines on *Th* gene, including exon 1, on HA-treated embryos, compared to vehicle-treated embryos, i.e., the percentages of modified bases were very similar (Figure 2B).

One limitation of bisulfite sequencing is that it does not allow discriminating between 5mC and 5hmC. Therefore, in order to evaluate both DNA modifications, we perform a MeDIP and hMeDIP, followed by qPCR in dissected VM (Figure 2A). Particularly, we focused on the intragenic regulatory regions of specification (*Lmx1a, Msx1, and Foxa2*) and differentiation (*Nurr1, Pitx3, and Th*) genes. We found that HA-injected embryos showed a significant decrease in 5mC at the exonic regions of the differentiation genes *Pitx3* and *Th*, compared to vehicle-treated embryos (Figure 2C). The 5hmC did not show significant changes on specification or differentiation genes neither in HA- nor in vehicle-treated embryos (Figure 2D).

### HA Treatment Is Associated With an Increase in the H3K9me3 and H3K27me3 Histone Modifications

Due to the histone post-translational modifications may also participate in the modulation of gene expression, we decided to study two particular histone modifications the H3K9 trimethylation (H3K9me3) and H327 trimethylation (H3K27me3). In order to evaluate the effect of HA treatment in the histone modifications H3K9me3 and H3K27me3 we performed a chromatin immunoprecipitation assay (ChIP) after the HA or vehicle treatment, and then evaluate the immunoprecipitated DNA by qPCR analyzing the above-mentioned regions of *Pitx3* (Figure 2E) and *Th* (Figure 2F) genes. We found that HA-injected embryos showed a significant increase of H3K9me3 and H3K27me3, in contrast with vehicle-treated embryos (Figure 2E,F).

### HA Injection Has a Long-Term Effect During the Formation of Midbrain Dopaminergic Pathways

**FIGURE 3:**
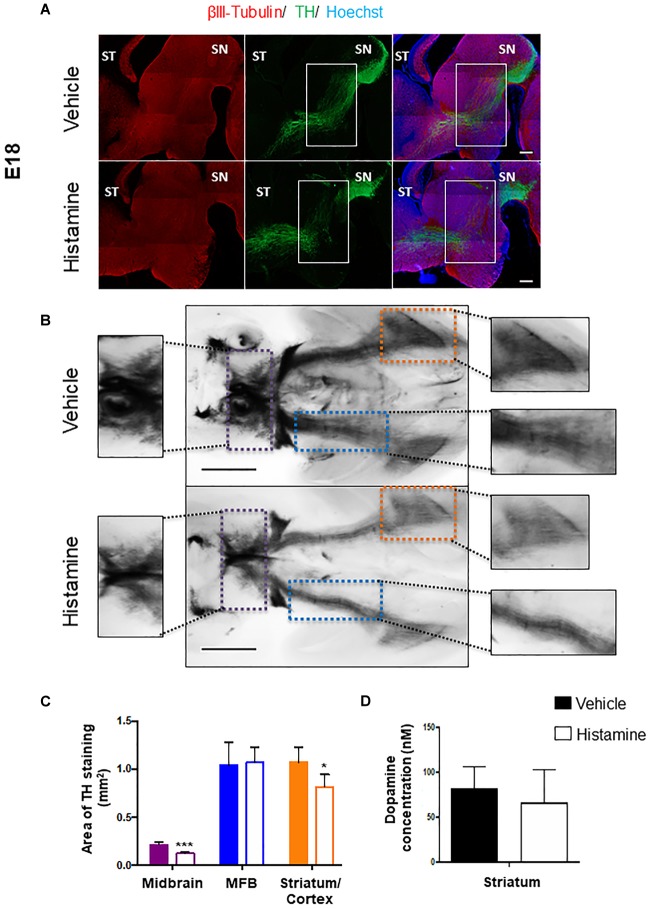
Analysis of the midbrain dopaminergic projection pathways at E18, after injection of HA at E12. **(A)** Immunohistochemistry for the limiting enzyme in DA synthesis, TH, analyzed together with β-III Tubulin, in sagittal brain sections 6 days after injection of vehicle or HA. Regions with differences between the two experimental conditions in the nigrostriatal pathway are marked with the white rectangles. ST, *striatum*; SN, *substantia nigra*. Scale bar: 500 μm. **(B)** Ventral views of the whole-mount TH immunostainings of E18 brains from embryos injected with vehicle or HA. The midbrain area within the dotted purple lines, the Medial Forebrain Bundle (MFB) area within the dotted blue lines and the Striatum/Cortex area within the dotted orange lines, are amplified in the intermediate panels, and were used to measure the area of TH staining in 4 independent experiments. Scale bar: 1 mm. **(C)** Quantification of TH staining area of Midbrain, MFB and Striatum/Cortex (this region also includes the Nucleus Accumbens). ^∗^*p* < 0.05 and ^∗∗∗^*p* < 0.005. **(D)** Dopamine content quantification in the striatum of vehicle- or HA-injected embryos by HPLC. The dissection of this area was performed at E18 making sure to remove the cerebral cortex and the basal part of the brain. Solid and empty bars apply to panels **C,D**.

We set out to investigate if the effect of HA on mDA 2-days after its injection has a long-term consequence, affecting the formation of nigrostriatal pathway. *In utero* injections of E12 embryos with either HA or vehicle were made, and the embryos were collected after 6 days, when normal development of the nigrostriatal pathway has reached the striatum. TH staining in sagittal sections or whole-mount immunohistochemistry was analyzed in E18 brains. A single injection of HA at early stages was enough to alter the formation of the nigrostriatal pathway; in vehicle-injected embryos, the MFB is apparently normal, whereas after HA administration a decrease in this bundle is observed and aberrant axons innervating the *thalamus* appeared (solid rectangles in Figure 3A). To comprehensively analyze the mesencephalic dopaminergic pathways, TH immunohistochemistry was performed in whole-brain mounts. Image analysis was used to quantify the indicated areas positive for TH within the dotted rectangles shown in Figure 3B. HA caused a significant reduction in the TH-positive area in the midbrain and the striatum/nucleus accumbens/cortex when compared to vehicle-injected embryos (Figure 3C). Also, the quantification of dopamine by HPLC assay in the striatal area shows a 19% non-significant reduction in HA injected animal compared to vehicle (Figure 3D). Together our data suggest that HA injection leads to a decrease in TH staining in the forebrain, which might result in lower dopamine release.

## Discussion

Brain development is initiated by formation of defined patterns in the neural ectoderm. As development proceeds, stem cell differentiation to neurons is defined by the action of morphogenic gradients and transcription factors, expressed in a temporal and sequential order (Gale and Li, 2008). In cultured cortical NSPC, HA has a neuronal-promoting action, involving up-regulation of proneural genes (Molina-Hernández and Velasco, 2008; Rodríguez-Martínez et al., 2012). However, HA administration *in vivo* diminishes proliferation of midbrain NSPC, down-regulating *Th* mRNA levels and the content of TH, Lmx1a, and Lmx1b assessed by western blot, 2 days after injection (Escobedo-Avila et al., 2014), but the mechanisms for these actions are just emerging (Molina-Hernández et al., 2013). In the current work, we administered embryos with either HA or vehicle, on a stage previous to the major production of DA neurons (Götz and Huttner, 2005), by ultrasound guided-injections. Injection of HA diminishes the presence of TH as well as the transcription factor Lmx1a in the VM.

Recently, studies about the influence of environment on epigenetics and its importance with regards to the expression of transcription factors during cell commitment have emerged. Regulation of the deposition of 5hmC in intragenic regions during progression of embryonic stem cells to NSPC is crucial for the development of mDA neurons (Kim et al., 2014). In dopaminergic neurons, methylation in one specific CpG site on the first exon of human *TH* gene down-regulates its transcription, allowing the binding of repressive transcription factors such as KAISO and NcoR and recruitment of HDACs, which might act as an additional modulator of *Th* expression (Arányi et al., 2005; van Heesbeen et al., 2013). We found that HA injection significantly decreased expression of early-differentiation genes (*Lmx1a* and *Msx1*) as well as in *Th*, a late canonical mDA differentiation gene.

Presence of 5mC and 5hmC within the exonic regions have been associated with up-regulation of gene expression, through different mechanisms (Hahn et al., 2013; Guo et al., 2014; Kim et al., 2014). Our findings reveal that injection of HA at early stages significantly diminish the percentage of enrichment of 5mC, but only on the differentiation genes *Pitx3* and *Th*. Although DNA methylation has been related to transcriptional repression, in recent years, some reports showed that 5mC within the gene body, specifically in exons, is associated to regulation of pre-mRNA splicing (Lev Maor et al., 2015). Other studies associated the gain of 5hmC at intergenic regions with transcriptional activation of neuronal differentiation genes (Hahn et al., 2013; Guo et al., 2014). We did not observe changes on 5hmC levels, indicating that HA induced gene repression independent of 5hmC, and suggesting that other epigenetic mechanisms participate in mDA gene regulation. Interestingly, many genes associated with neuronal differentiation can be negatively regulated by decreasing 5mC and a gain in histone repressive modifications, such as H3K27me3, at their regulatory regions (Hahn et al., 2013).

During mDA differentiation, epigenetic changes occur in cultured VM NSCP: Nurr1-controlled dopaminergic gene expression was precluded by the interaction of Nurr1 with CoREST and HDAC1, a complex that caused deacetylation of these promoters. Forced expression of Foxa2 allowed the formation of Nurr1-Foxa2 complexes with concomitant acetylation of H3, associated to open chromatin, in *Th* and *Dat* promoters (Yi et al., 2014). *In vitro* expansion of VM NSCP caused decreased dopaminergic differentiation, which was associated with hypoacetylation on H3 and H4 and increased H3K27me3 in the Foxa2 promoter, as well as globally. Inhibition of HDAC activity up-regulates *Foxa2* transcripts, caused H3 acetylation, decreased methylation of H3K27 and restored dopaminergic induction (Bang et al., 2015). We observed significant increases in the repressive marks H3K27me3 and H3K9me3 in *Pitx3* and *Th* genes, although significant down-regulation was observed only in *Th*. Similar observations were recently reported by Hong et al. (2019), where they manipulated *in vitro* the levels of H3K27me3 in the promoter region of *Th*: treatment with EPZ005687, an inhibitor of histone methylase EZH2 reduces the levels of H3K27me3 and leads to an increase in TH levels; conversely, addition of GSK-J1, which affects the demethylase activity of Jmjd3, augmented H3K27me3 and decreased TH protein levels (Hong et al., 2019). Our data points that not only the H3K27me3 is involved in the regulation of *Th*, but also H3K9me3 participate.

We studied the long-term consequence of deregulation of such genes, in the formation of the MFB, and found that injection of HA caused disrupted and miss-oriented dopaminergic fibers in sagittal sections. Further, a significant reduction in TH-positive staining was found in the MB and the striatum/cortex after HA treatment, in whole-brain mounts. However, we did not observe a complete deterioration of the nigro-striatal, mesolimbic and mesocortical pathways, probably due to the plasticity of the developing brain; accordingly, dopamine level in the striatum were not reduced by HA. In newborn rats injected with 6-hydroxydopamine to degenerate mDA neurons, there is an increase in serotonergic projections (Snyder et al., 1986) and lesioned animals were hyperactive (Miller et al., 1981; Zhang et al., 2001; Avale et al., 2004). It will be of interest to analyze if embryos injected with HA at E12 show changes in locomotor activity at postnatal stages.

**FIGURE 4:**
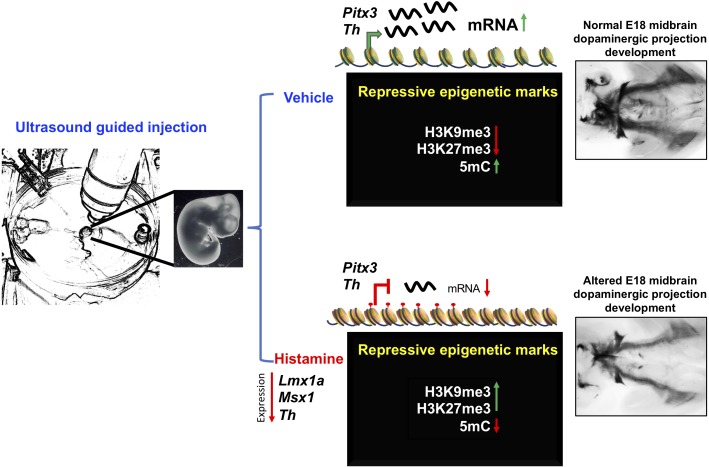
Proposed mechanism of HA action on the regulation of late mDA differentiation genes. Intrauterine administration of HA into the MB of E12 rat embryos, decreases the expression of specification and differentiation dopaminergic genes, such as *Lmx1a*, *Msx1*, and *Th*. The decrease in *Th* expression induced by HA was correlated with the gain of histone repressive marks, H3K27me3 and H3K9me3, together with the loss of 5mC at intergenic regulatory regions. The down-regulation of these key dopaminergic differentiation genes, particularly *Th*, resulted on the alteration of the midbrain dopaminergic projection development at E18.

The precise mechanisms that link HA and the epigenetic changes reported here deserve further investigation. In cultured VM-NSPC, it has been shown that membrane depolarization and calcium influx is correlated with higher acetylation of Histone 3, trimethylation of K4 of H3 with reductions in the H3K9me3 and H3K27me3 marks (He et al., 2011). HA triggers intracellular calcium increases after receptor activation in VM-NSPC, through production of IP3 and Diacylglycerol by Phospholipase C, but the effects reported here are in the opposite direction: addition of the transcriptional repressive H3K9me3 and H3K27me3; additional signaling transduction mechanism to explore include Phospholipase A2/arachidonic acid production and nitric oxide production/cGMP increases (Molina-Hernández et al., 2012). On the other hand, the participation of the transient population of histaminergic neurons of the raphe nucleus or in the hypothalamus (Vanhala et al., 1994), and the activation of microglia after HA administration, as observed in adult mice (Rocha et al., 2016), cannot be ruled out in our experiments; therefore, it will be interesting to determine their roles in future experiments by ablating HA-producing neurons or microglial cells, respectively. All together, the data reported here describe molecular effects of HA on dopaminergic midbrain development. The reduction in expression of dopaminergic genes correlates with gain of the repressive modifications H3K9me3 and H3K27me3 and the loss of DNA methylation at intergenic regions in *Th*. Moreover, HA induced long-term alterations on mDA neurons, as shown in Figure 4. Our results suggest that HA promotes an epigenetic change related to significant reductions in the expression of important dopaminergic genes. Such alterations result in anatomical dysregulation and decreased TH staining in the dopaminergic neurons targets.

In conclusion, this study contributes to the understanding of the molecular actions of HA on mDA development, as well as chromatin structure regulation mediated by extracellular stimuli, during NPSC commitment in the midbrain; such knowledge may contribute to establish different roles of HA during brain development.

## Data Availability

The datasets generated for this study are available on request to the corresponding author.

## Ethics Statement

This study was carried out in accordance with Mexican guidelines and following recommendations of Instituto de Fisiologia Celular, UNAM Animal Care and Use Committee, which approved this protocol.

## Author Contributions

FV-R wrote the manuscript, analyzed the data and performed the majority of experiments, including epigenetics, whole mount stainings and dopamine quantification. LG-C and RG-B performed RT-qPCR and ChIP assays, contributed to the design of experiments and discussion of results. IE-A helped to perform intrauterine injections, data analysis and participated on discussion of results. DC-P contributed to set up techniques, performed results analysis and discussed results. AL-O and LR participated in dopamine quantification by HPLC. IV and ES-R contributed to the experimental design and supervision, reviewed the manuscript, and obtained funding. All authors read and approved the final manuscript.

## Conflict of Interest Statement

The authors declare that the research was conducted in the absence of any commercial or financial relationships that could be construed as a potential conflict of interest.
